# Modeling K12 Teachers’ Online Teaching Competency and Its Predictive Relationship with Performance—A Mixed-Methods Study Based on Behavioral Event Interviews

**DOI:** 10.3390/bs15050628

**Published:** 2025-05-05

**Authors:** Jun Tian, Wenhui Tian

**Affiliations:** 1 School of Information Technology in Education, South China Normal University, Guangzhou 510631, China; tjun@m.scnu.edu.cn; 2 School of Computer and Information Engineering, Hubei Normal University, Huangshi 435002, China

**Keywords:** online teaching, competence, behavioral event interview, performance, predictive relationship, K12 teachers

## Abstract

This study constructs and validates a multidimensional online teaching competency model for K12 teachers through an integrated mixed-methods design. Combining behavioral event interviews (*n* = 38) with large-scale psychometric evaluation (*n* = 4378), we identified six hierarchically organized competency dimensions encompassing 29 measurable elements. The model differentiates between 12 discriminative competencies and 17 baseline competencies, further categorized into explicit (knowledge, technical, instructional, management) and implicit (achievement orientation, individual traits) dimensions. Exploratory and confirmatory analyses validated the model’s robust multidimensional structure (CFI = 0.923, TLI = 0.914, RMSEA = 0.042). Structural equation modeling revealed significant competency-performance linkages, with 10 of 12 hypothesized paths attaining statistical significance (*p* < 0.05). Management competencies emerged as the strongest predictor of both process (β = 0.37) and outcome performance (β = 0.29), followed by instructional competencies (β = 0.31 and 0.24 respectively). The model provides empirically grounded guidance for developing online teaching norms, competency-based teacher training programs, and performance evaluation systems.

## 1. Introduction

The global educational landscape has undergone unprecedented digital acceleration since 2020, with over 89% of OECD countries institutionalizing national online learning platforms by 2021 (OECD, 2021). Within this worldwide transformation, China’s 2022 National Smart Education Platform launch represents a strategically scaled implementation, serving as an empirical testbed for competency model validation under high-stakes conditions. This platform, integrated across 32 provincial systems, now supports 18.3 million K12 teachers, making it a critical context for studying systemic digital pedagogy transitions. The reform of teaching models supported by digital resources has been continuously advancing, leading to changes in the teaching and learning environment, classroom structures, and teaching methods. A new educational ecosystem that integrates both online and face-to-face education has gradually emerged (Chen, 2020). This also placed new demands on K12 teachers, making online teaching a norm in their daily work.

Although the investment in infrastructure has increased in various countries, teacher readiness remains a universal bottleneck. UNESCO’s 2022 Global Education Monitoring Report identifies “competency gaps in technology-mediated instruction” as the top barrier to equitable digital learning in 76% of surveyed countries (UNESCO, 2022). In China, there are misunderstandings about online teaching (X. P. Wang, 2020), mirroring issues observed in India’s DIKSHA platform (Kumar & Sharma, 2021) and Brazil’s Aula Digital (Araújo et al., 2021). For instance, some equate it with live teaching, recorded teaching, or simply transferring face-to-face teaching methods online without adaptation. Zhao et al. (2022) surveyed 3107 K12 teachers and found that the most concerning issues in practice remain the operation of online teaching platforms and resource application. The overall understanding and adaptability to online teaching are at a moderate level, with significant urban–rural disparities.

Additionally, both pre-service and in-service stages of K12 teacher training lack systematic online teaching capability development (Yang & Zhang, 2020). During the pre-service stage, there are some courses such as “Foundations of Education” and “Multimedia Technology and Application” in training programs, as well as practicum and internship components. However, these focus discretely on pedagogy or the application of resource creation tools and subject tools, without sufficient emphasis on designing instructional activities, selecting teaching strategies, and designing instructional evaluations in online environments. During the in-service stage, training related to online teaching for K12 teachers still exhibits a “skill-oriented” bias, rarely addressing the diverse competencies required during online teaching, such as organizing learning activities, designing interactions, and analyzing student performance based on data. Fu (2020) surveyed 7111 teachers and found that most trainings were either too theoretical to be practically implemented or too technical, assuming that mastering the use of teaching software tools suffices for effective online teaching.

Furthermore, many schools or regions still evaluate such performance solely through student exam scores—a reductionist approach that neglects critical multidimensional aspects, including systematic activity design, collaborative learning dynamics, and the cultivation of students’ multiple intelligences during online instruction, thereby underscoring the urgency for evidence-based evaluation frameworks. This study conceptualizes online teaching performance as teaching effectiveness that encompasses two dimensions: process performance (e.g., learner engagement efficiency, instructional interaction quality) and outcome performance (e.g., academic achievements, skill development; Rahmatullah, 2016; Gökdaş et al., 2024).

Therefore, the digital transformation of education demands reimagined teacher competencies, which is a challenge that is both context-specific and globally resonant. In order to solve the problems of K12 online teaching standardization, online teaching training systematization, and online teaching evaluation scientifically, it is necessary to use scientific methods to construct an online teaching competence model. This study aims to answer the following research questions:

RQ1: What are the characteristics of K12 teachers’ online teaching competence?

RQ2: What is the structure of the competency model composed of these characteristics?

RQ3: What is the relationship between the competency characteristics in the model and the prediction of online teaching performance?

## 2. Literature Review

Competence is not merely an ability but also a qualification or prerequisite for engaging in a particular job. In specific work contexts, it refers more to comprehensive capabilities rather than basic problem solving, encompassing not only explicit knowledge and skills but also implicit traits, qualities, values, and motivations. For this reason, the competency model of teachers in specific work or job contexts has increasingly gained attention from researchers.

### 2.1. The Competence of K12 Teachers

#### 2.1.1. Theoretical Foundations

Research on competency stems from the continuous specialization of social division of labor following the Industrial Revolution, and has permeated various fields such as management, economics, and education. McClelland (1973) introduced the concept of “competency”, defining it as “the knowledge, skills, abilities, traits, or motives that are directly related to job performance or important outcomes in the process”. Building on this foundation, Spencer (1993) proposed the classic theoretical model of competency, the “iceberg model”, which posits that above the surface, visible traits include skills and knowledge, which are easily acquired and changed; below the surface, invisible deep-seated competencies, such as self-concept, social roles, and motivations, play a decisive role in behavior and performance.

#### 2.1.2. Applied Frameworks in Varied Contexts

Emerging from these theoretical foundations, empirical investigations of teacher competency models coalesce into two thematic paradigms.

Firstly, process-oriented frameworks emphasize instructional workflows. Danielson et al. (2024) used the teaching business process as a clue and proposed a four-dimensional model, including planning and preparation, classroom environment monitoring, teaching, and professional responsibility. Richey et al. (2001) proposed a teacher competency model for instructional design consisting of four dimensions and 19 competency characteristics, including professional basic abilities, planning and analysis, design and development, implementation, and management. Koszalka et al. (2013) also focused on instructional design and developed a teacher competency model including professional basic abilities, planning and analysis, design and development, implementation, and management. Ferrández-Berrueco and Sánchez-Tarazaga (2014) believed that teachers should possess competencies in four areas: subject matter, methodological, social, and personal abilities.

Secondly, scenario-specific adaptations address contextualized pedagogical demands. Many scholars have also conducted research on teacher competency for different educational stages, positions, or teaching scenarios. For example, X. L. Luo (2010) interviewed 28 middle school teachers and supplemented with open questionnaires to construct a middle school teacher competency model consisting of nine competency characteristic groups. Yuan et al. (2021) constructed a home-school cooperation competency model for K12 teachers, including three levels: home-school cooperation knowledge and skills, attitudes and values, and personality and achievement motivation. X. Wang et al. (2024), focusing on interdisciplinary teaching readiness (ITR), confirmed that ITR consists of three factors (including interdisciplinary teaching knowledge structure readiness, interdisciplinary teaching skills readiness, and interdisciplinary teaching attitudes readiness) and 24 items using item analysis and critical ratio method. Zhou et al. (2024) investigated the significance of improving cross-cultural communicative competence (CCC) in undergraduate English instruction.

#### 2.1.3. International Standards

Additionally, some countries and organizations have also proposed relevant models to promote professional development among teachers. For instance, UNESCO (2022) established ICT competency standards for teachers from six aspects: understanding ICT, curriculum and assessment, pedagogy, application of digital skills, organization and management, and teacher professional learning. The Australian Institute for Teaching and School Leadership published the *Australian Professional Standards for Teachers* in 2011 and revised them in 2018. AITSL (2018) includes three domains (knowledge, practice, and engagement) and seven standards. The Teachers College of Columbia University (2015) developed a global competence certificate, including cognitive skills (critical thinking, creative thinking, comparative and reasoning abilities, information discernment and judgment capabilities, and expression of viewpoints) as well as action skills (digital literacy, dialogue abilities, translating ideas into actions, communication in dialogues, collaborative cooperation, shared responsibility, and sharing of outcomes).

### 2.2. The Competence for Online Teaching

Research on online teaching competency has evolved along two distinct yet complementary trajectories. The first strand focuses on conceptualizing the multidimensional roles and capabilities required for remote instruction, while the second centers on establishing standardized frameworks for digital education competence.

#### 2.2.1. The Conceptual Development

The conceptual research trajectory reveals a progressive expansion of understanding teacher roles in digital environments. Early foundational work by Thach and Murphy (1995) established 11 critical roles ranging from instructional design to evaluation expertise, accompanied by 10 core capabilities. Subsequent studies progressively refined these dimensions: Williams (2003) expanded the role taxonomy to 13 positions, including technical specialists and instructional supporters, while Goodyear et al. (2001) introduced communication dynamics through 23 capability–task pairings. This conceptual evolution continued through Kabilan’s (2004) emphasis on motivational and ideological dimensions, Dabbagh’s (2003) nine-role interaction model, and Alvarez et al.’s (2009) five-category system integrating social and managerial competencies. More recent contributions, such as Ally (2019), have synthesized these elements into comprehensive nine-domain models encompassing both pedagogical and technological dimensions.

The global shift towards prioritizing information and communication technology has underscored the pressing need to bolster and cultivate digital competence within educational contexts (Guillén-Gámez et al., 2021). Basilotta-Gómez-Pablos et al. (2022) reviewed studies on the digital competence of university teachers, revealing a dominant focus on self-assessment and the need for more practical training programs. Bilbao Aiastui et al. (2021) and De la Calle et al. (2021) have further explored the integration of digital skills in education, highlighting the need for continued research and development in this area.

#### 2.2.2. Establishing Standardized Frameworks

Parallel to these conceptual developments, institutional standardization efforts have emerged. The “EU Framework for Digital Education Competence of Teachers” proposed in 2017 includes a model of teacher digital competence with six domains and 22 capabilities, such as professional engagement, digital resources, teaching and learning, assessment, empowering learners, and promoting learners (Redecker, 2017). The International Society for Technology in Education in the United States also revised the “National Educational Technology Standards for Teachers” in 2017, categorizing teachers into seven functional roles, including learners, leaders, citizens, collaborators, designers, facilitators, and analysts (Feng et al., 2018).

Despite these advancements, three critical gaps emerge from the existing scholarship. First, the existing research in the field of online teaching competency has focused more on the rapidly developing applications and advancements in higher education, distance education, with less attention paid to K12 education. Second, current models insufficiently account for the blended learning realities of contemporary K12 education, where online–offline integration requires distinct competency profiles differing from pure distance education models. Third, there’s limited empirical validation of proposed frameworks in actual K12 classroom implementations (Bilbao Aiastui et al., 2021; De la Calle et al., 2021). These lacunae highlight the need for a purpose-built competency framework addressing the unique requirements of K12 teachers navigating hybrid educational landscapes.

### 2.3. The Relationship Between Competency and Performance

The competency-performance nexus has been systematically examined across disciplines through three primary research lenses: empirical validation studies, process-outcome differentiation analyses, and contextual adaptation investigations.

#### 2.3.1. Empirical Validation Studies

Empirical validation studies, covering a wide range of topics, including education, medicine, and business management, have established robust quantitative foundations for this relationship. This study focuses on the relationship between teachers’ competencies and teaching performance in an online teaching environment. Syarif and Angger (2017) used quantitative research methods to study the relationship between professional competency and performance among public primary school teachers in Central Java, India, and confirmed a significant positive correlation between primary school teachers’ competency and performance (r = 0.979). Wahyuddin (2016) also studied Indian teachers and investigated the relationship between teacher competence and emotional intelligence through descriptive and inferential analysis. The results showed that there is an association between teacher competence and teacher performance, and there is also an association between emotional intelligence and teacher performance.

Both process-based performance and outcome-based performance have been proven to be related to teachers’ competencies. Rahmatullah (2016), in exploring the correlation between learning effectiveness (focused on the learning process) and teacher competence, confirmed that there is a high correlation between teacher competence and both learning efficacy and teacher performance; and there is a relationship between learning efficacy and teacher performance. Tabassum et al. (2020) reached a similar conclusion in their investigation of the relationship between social competence and academic performance (focused on outcomes) among college students. The sample included 4708 participants from different universities in Pakistan. The data analytics results showed that the relationship between academic performance and social competence was significant. Similarly, the social skills, cognitive skills, and interpersonal skills of social competence had a significant impact on academic performance.

#### 2.3.2. Contextual Adaptation Research

Contextual adaptation research introduces organizational moderators into this relationship. Junior et al. (2017) believes that professional competence includes knowledge, skills, abilities, traits, and behaviors, and in a survey exploring the relationship between employee competence and job performance in public organizations, it was found that the support provided by management has a significant impact on employee performance, and that different structures of competence have different effects on employee performance.

With the rapid development of digital technologies, teachers need to be able to use these technologies effectively and develop students’ digital skills. Gökdaş et al. (2024) found through a comprehensive literature analysis that among the seven factors identified as influencing teachers’ digital competence, 26.34% of the literature focused on the link between teachers’ digital competences and student performance, demonstrating that enhanced digital skills positively impact student outcomes.

Three critical syntheses emerge from this body of research. First, the competence-performance relationship demonstrates cross-contextual validity through replicable effect sizes. Second, performance measurement specificity determines observed effect magnitudes-composite measures yield stronger correlations than single indicators. Third, digital transformation introduces new mediating variables, particularly technological adaptability and student-facing digital literacies.

These syntheses justify our operational definition of performance as encompassing both process metrics (learning habits, self-learning ability, etc.) and outcome indicators (learning achievements, professional development, etc.).

## 3. Methodology

**Figure 1:**
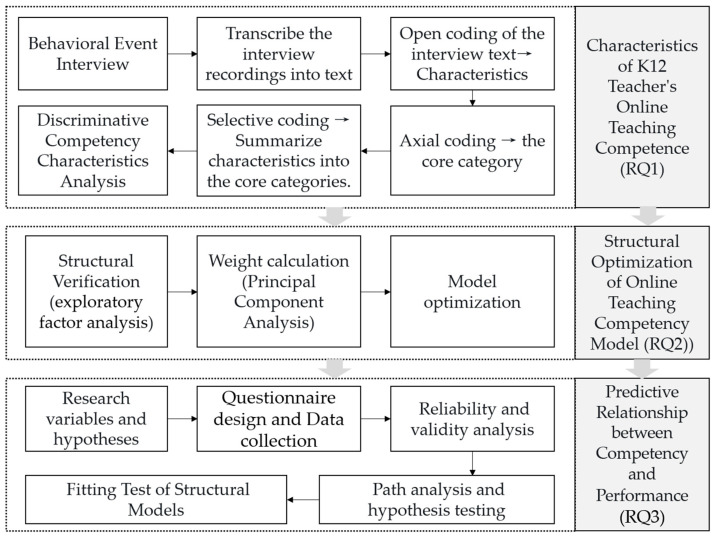
Research design.

To address the three research questions outlined above, the study is divided into three phases, each corresponding to a distinct sub-study (Figure 1).

### 3.1. Behavioral Event Interview (BEI) for RQ1

This sub-study aims to explore the characteristics of the online teaching competency model using the behavioral event interview (BEI) method. This approach involves a time-compressed observation of actions, where respondents are asked to describe in detail key instances of effective and ineffective work in their professional activities. Through coding and analysis, these instances are broken down into specific behaviors, thereby identifying the competencies required for a particular job or position. The procedure is structured as follows.

First, interviews were conducted with teachers. A random sample of 25 teachers was selected, comprising 13 high-performing teachers and 12 ordinary-performing teachers from the same schools. Face-to-face interviews were conducted and audio-recorded.

Second, transcription and open coding were conducted. The recorded interviews were transcribed verbatim. Open coding was applied to the transcripts to extract competency-related characteristics articulated by the teachers.

Third, axial coding was conducted. The outcomes of open coding were analyzed to categorize emergent competency characteristics into core genera.

Finally, selective coding was conducted. A core genus was selected to integrate the characteristics derived from open coding, resulting in a preliminary competency model that maps characteristics to their respective dimensions.

### 3.2. EFA and PCA for RQ2

This phase employed exploratory factor analysis (EFA) and principal component analysis (PCA) to validate and optimize the model.

The first step was questionnaire development and data collection. The competency characteristics identified in Phase 1 were operationalized into a questionnaire distributed to K12 teachers. Then, an exploratory factor analysis (EFA) was conducted to verify the alignment of characteristics with their hypothesized core dimensions. Moreover, weight assignment via principal component analysis (PCA) was utilized to calculate the relative weights of each competency feature within the model. This process yielded a revised competency model with weighted characteristics’ contributions.

### 3.3. SEM for RQ3

The relationship between competency features and teaching performance was examined using structural equation modeling (SEM).

Firstly, teaching performance indicators were defined based on a literature review, followed by the formulation of research hypotheses. Next, a questionnaire targeting performance indicators was administered, and the collected data underwent reliability and validity analyses. Furthermore, SEM-based path analysis was performed to test the hypothesized relationships between the competency characteristics and teaching performance. Finally, the predictive structural model linking competency characteristics to teaching performance was statistically validated.

## 4. Characteristics of K12 Teachers’ Online Teaching Competence (RQ1)

Competency modeling identifies the critical characteristics that individuals require to excel in specific professional roles (Wiyanah et al., 2021). Addressing RQ1, this study employs the BEI method to systematically examine the distinctive competencies underlying effective online instruction. The BEI approach is particularly suited for this investigation as it: (1) captures actual teaching behaviors rather than self-reported perceptions, (2) has demonstrated effectiveness in educational competency research, such as Wiyanah et al. (2021), which takes this approach, emphasizing process and constructing a model of teachers’ English teaching competence for presentation, practice, and production (PPP), and (3) enables differentiation between baseline and exceptional performance. Focusing specifically on K12 online teaching contexts, our analysis aims to establish a comprehensive competency framework that reflects both the unique demands of digital pedagogy and the developmental needs of teachers.

### 4.1. Interview Outline Design

To better guide respondents in describing the most successful and regrettable events in a specific job and to uncover deeper behavioral details, this study utilizes the STAR tool. The STAR tool designs the interview outline from four aspects: situation, task, action, and result, as shown in Table 1.

### 4.2. Interview Implementation and Reliability Analysis

The respondents were divided into a high-performing group and an average-performing group. Both groups were required to have over 30 h of online teaching experience. Additionally, the high-performing group was defined by meeting at least one of the following criteria: (1) holding distinguished teaching titles such as “key subject teacher” or “academic discipline leader”; (2) achieving provincial-level or higher awards in significant educational initiatives (e.g., the “One Teacher, One Exemplary Lesson” national campaign); or (3) attaining senior professional titles (Level 1 or advanced certification). The average-performing group consisted of teachers randomly selected from the same schools as high-performing participants, matching the minimum 30 h online teaching experience requirement. The interview process employed a single-blind design, ensuring participants remained unaware of their group assignment throughout the study.

A total of 13 teachers were interviewed in the high-performance group, with an average teaching experience of 16.8 years and an average of 80.7 h of online teaching; 12 teachers in the general-performance group, with an average teaching experience of 9.04 years and an average of 47.9 h of online teaching. The respondents did not know to which group they belonged in advance, and the study utilized a single-blind design.

In order to improve the reliability and validity, before the formal interview, three teachers from a primary school in Dongguan City, Guangdong Province, were sampled for pre-interview, and the statements and questions reported by the respondents as being difficult were corrected. The formal interviews were completed during four teacher trainings organized by the Hubei Audio-Visual Education Museum. During the interview, the interviewees were mainly guided to describe three key events of success and regret in online teaching practice in an appropriate order based on the interview outline. After the interview, “iFLYTEK Listening Software” (3.0) was used to transform the interview recordings into text. After screening out one sample that failed automatic speech recognition due to dialect and two samples that were biased from the interview outline, this study ultimately compiled 22 interview transcripts totaling 178,000 words, yielding an average of 8078 words per participant (calculated by dividing total word count by 22 respondents). This average notably surpasses the 8000-word threshold for sample stability in BEI data established by Shi et al. (2002).

The study selects classification consistency and coding reliability coefficient to measure its reliability. Category agreement (CA) considers the degree of consistency of coding classification of the same interview text content by multiple coders, and its calculation formula is: CA = 2S/(T1 + T2) (Smith, 1992), where S refers to the number of coders with the same coding classification, and T1 and T2 represent the total number of coders of two coders, respectively. Coding reliability coefficient (R) is compound reliability, and the calculation formula is: R = (n × CA)/[1 + (n − 1) × CA] (Dong, 2019), where n is the number of coders.

In this study, the classification consistency and coding reliability calculation results of the two coders for 22 interview texts are as follows: the maximum value of classification consistency CA is 0.938, the minimum value is 0.551, the maximum value of coding reliability coefficient is 0.955, and the minimum value is 0.711; the overall classification consistency is 0.655, and the coding reliability coefficient is 0.787, indicating that the consistency of the two coders is at a good level and the reliability is high. The coding results can be further analyzed statistically.

### 4.3. Open Encoding

The coding and analysis of interview text is an important process to draw research conclusions based on BEI. The coding process follows the logical sequence of grounded theory’s “open coding-axis coding-selective coding”.

Open coding is the process of analyzing, inspecting, conceptualizing, and comparing data. The purpose is to discover and name concept categories from interview text materials. In this study, NVivo software 11.0 was used for open coding, and coding nodes were set up from three aspects.

First, data were extracted from 23 studies that were closely related to “online teaching competence” selected in the literature analysis and classified according to the three levels of skills and knowledge, attitudes and values, and traits and motivations using the “iceberg model”. After merging similar or duplicate items, an initial set of 32 alternative competency characteristics was formed, which served as a reference node for open-ended coding. Second, names were self-created, using the coder’s understanding of the views mentioned in the interview text, which can reflect the meaning of the text as nodes. Third, the real coding was conducted, and words were extracted from the utterances used by the respondents themselves as coding nodes.

When coding, if there were characteristic items that were not available in the initial set, they were directly added to the coding nodes. After the initial coding, the text was reviewed, the coding was checked, all existing evidence supporting a categorized coding was confirmed, and it was determined whether they intersect or include each other. Finally, characteristic items such as “ individual learning feedback” and “group learning feedback” that did not appear in the initial set were added. “Distance immediate response” and “critical thinking,” which existed in the initial set but were not mentioned in the interview, were deleted, resulting in a list of 32 open coding nodes, as shown in Table 2.

### 4.4. Axis Coding and Selective Coding

The main axis coding established the relationship between concept genera through deduction and induction, and connected the main and secondary concept genera to form a two-level coding. Selective coding extracted a “core genus” that can briefly explain all the phenomena through the genera and relationships developed by the first two levels of coding. Through open coding analysis, we identified 32 characteristic items that were systematically categorized into six core dimensions: (1) knowledge characteristics, (2) technical characteristics, (3) instructional characteristics, (4) management characteristics, (5) achievement characteristics, and (6) individual traits. Consistent with McClelland’s (1973) iceberg model, the first four dimensions (knowledge, technical, instructional, and management) represent explicitly observable competencies and trainable skills at the “surface level”. The remaining two dimensions (achievement characteristics and individual traits) constitute implicit competencies, reflecting deeper, less observable attributes that underlie superior performance. In this process, pedagogical knowledge and psychological knowledge were merged into pedagogical and psychological knowledge (PPK), individual learning feedback and group learning feedback were merged into learning feedback (LF), and autonomous learning ability was merged into autonomous development consciousness, and 29 characteristic items were obtained, as shown in Table 3.

### 4.5. Discriminative Competency Characteristics Analysis

For the coded data, a further differential analysis was carried out to test the content validity of the code on the one hand, and which competency characteristics were discriminative on the other hand. Therefore, the average grade score with better stability in relation to the length of the interview text (Shi et al., 2002) was selected, and the difference analysis was performed on 30 coding nodes to evaluate its validity and explore the discriminative competency characteristics (Hair et al., 1995).

In order to identify the characteristic items that can distinguish between high performance and ordinary performance, an independent sample *t*-test was conducted on the average grade scores of competency characteristics of teachers in the high-performance group and the ordinary-performance group. The average grade scores refer to the evaluation process, where, after initially coding the interview transcripts to identify competency items in the previous step, coders re-analyzed the textual data to assess participants’ behavioral performance across each established competency dimension.

Data analytics found that there were significant differences in 12 competency characteristics between the two groups (Table 4). They are fused knowledge (t = 1.975, *p* = 0.047 *), technology adaptation (t = 3.304, *p* = 0.003 ***), collaborative teaching ability (t = 3.285, *p* = 0.003 **), data-based learning situation analysis (t = 2.827, *p* = 0.027 *), online learning evaluation (t = 2.707, *p* = 0.031 **), learner retention (t = 3.700, *p* = 0.003 **), distance emotional perception (t = 2.529, *p* = 0.019 **), learning feedback (t = 3.247, *p* = 0.004 **), community thinking (t = 3.673, *p* = 0.000 ***), achievement motivation (t = 4.167, *p* = 0.000 ***), teaching self-efficacy (t = 4.453, *p* = 0.000 ***), and communication skills (t = 4.246, *p* = 0.000 ***). These characteristics can distinguish between excellent and ordinary teachers and are classified as discriminative competencies, while others are classified as benchmark competencies.

The identified discriminative competencies refer to those qualitatively distinct capabilities that statistically differentiate high-performing educators from their peers. In contrast, benchmark competencies encompass threshold qualifications required for normal occupational adequacy. This dichotomy aligns with McClelland’s (1973) iceberg model, where discriminative competencies reside in the submerged “differentiating factors” layer, whereas benchmark competencies constitute the visible “baseline requirements” (Boyatzis, 2008).

**Figure 2:**
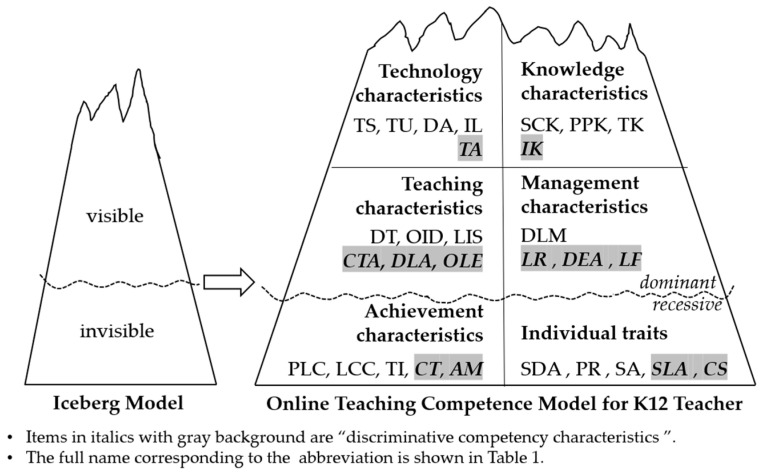
The initial model of online teaching competence of K12 teachers.

Combined with the above coding and analysis results, an initial model of online teaching competency for primary and secondary school teachers was developed, which includes 29 competency characteristics in six core categories, as shown in Figure 2. Among them, knowledge characteristics, technical characteristics, teaching characteristics, and management characteristics belong to explicit characteristics, and achievement characteristics and personal characteristics belong to recessive characteristics. This includes two categories. One is benchmark competency; that is, the common competency characteristics in online teaching, which are the basic requirements for online teaching work, with a total of 17 items. The other is discriminative competency; that is, the competency characteristics that can distinguish high-performance teachers from ordinary performance teachers, with certain discrimination ability, a total of 12 items.

## 5. Structural Optimization of Online Teaching Competency Model (RQ2)

Building on our initial competency characterization (RQ1), we now address RQ2: What is the structure of the competency model composed of these characteristics? To establish both empirical validity and theoretical coherence, this phase employs factor analysis, principal component analysis, and model fitting techniques. These methods serve three critical functions: (1) verifying the statistical robustness of individual competency items identified through BEI and mitigating potential researcher bias inherent in qualitative interpretations, (2) optimizing the dimensional aggregation of these features, and (3) clarifying their weight distribution in the competency model.

Delcker et al. (2025) constructed a six-dimensional artificial intelligence competency model for teachers through a survey of 480 teachers in vocational schools. Raúl González-Fernández et al. (2024) conducted quantitative data analytics using the Logit model through a questionnaire survey of more than 100 teachers. The results show that teachers should possess competencies including leadership, research, sharing and reflection, collaboration and communication, and digital and innovation. This study’s data source is a questionnaire survey of 4378 K12 teachers sampled from 23 districts (counties) in China (Table 5). The questionnaire was distributed online through official education administration work groups, where teachers voluntarily filled it out to become participants. The survey platform used was Wenjuanxing (https://www.wjx.cn, accessed on 16 October 2024).

There are two sources of the scale for the core variables in the questionnaire: one is the mature scale tested by reliability and validity in the existing research, which was revised and quoted according to the research context; the other is the unavailable scale, which was compiled according to the manifestation and connotation of the feature items in the behavioral event. In the recycled data, the internal consistency coefficient (Cronbach Alpha) of each dimension is greater than 0.7, and the reliability is good.

### 5.1. Structural Verification

In order to verify whether it is reasonable to divide the online teaching competence of K12 teachers into six core categories and 29 characteristic items in the initial model, an exploratory factor analysis was carried out on the data. First, the results of Bartlett spherical and KMO tests showed that the KMO value was 0.878, greater than 0.6, and the Bartlett spherical test value (approximate chi-square) was 8639.971 (Sig. = 0.000), which reached the standard of scholar Hair et al. (1995). Second, the principal component factor extraction method was selected, and the maximum variance method was rotated; a total of six common factors were extracted, and the cumulative variance interpretation rate was 67.27%. Overall, the results of exploratory factor analysis were good and consistent with the six feature groups in the initial model.

In order to further explore the corresponding relationship between the six factors and the items, and in order to verify whether each second-order competency characteristic item belongs to the six core genera constructed by qualitative analysis, the rotated component matrix results were analyzed, and the results show that each competency characteristic factor has no double load phenomenon, and all the item factor loads are greater than 0.4. That is to say, it is confirmed that the online teaching competency model of K12 teachers is a multi-dimensional hierarchical structure, composed of six dimensions: technical characteristics, knowledge characteristics, teaching characteristics, management characteristics, achievement characteristics, and personal characteristics. The attribution of competency characteristic factors represented by most of the items is consistent with the initial model.

However, “learning feedback” is attributed to management characteristics in the initial model, while exploratory factor analysis shows that it should be attributed to teaching characteristics. “Data-based learning analysis” and “online learning evaluation” are also not attributed to teaching characteristics as expected by the initial model, but to management characteristics. “Self-development consciousness” is attributed to individual traits in the initial model, and is adjusted to achievement characteristics here. Therefore, the initial model needs to be adjusted according to the results of exploratory analysis.

### 5.2. Weight Calculation

The methods of weight calculation usually include principal component analysis, AHP analytic hierarchy process, entropy method, etc. To quantitatively refine the initial competency model derived from qualitative research, principal component analysis (PCA) was selected for weight calculation due to its ability to: (1) objectively determine weights based on variance maximization, (2) reduce subjective bias compared to expert-dependent methods (e.g., AHP), and (3) handle multidimensional competency indicators through data-driven dimension reduction.

The calculation process is divided into three steps. First, SPSS 25.0 is used for standardization and principal component analysis. The results show that the principal component characteristic roots of the six common factors are 15.323, 8.446, 4.967, 3.707, 2.151, and 1.840, respectively. Second, the coefficients of each characteristic term in the linear combination of principal components are calculated, that is, the weights of each factor. Third, the scores of each variable in the comprehensive model are calculated, and the weights are obtained by normalization.

Table 6 presents the computed weight distribution across six competency dimensions, with teaching characteristics (w = 0.25) and management characteristics (w = 0.24) emerging as the most critical components, followed by achievement characteristics (w = 0.16). The remaining dimensions show progressively lower weights: technical characteristics (0.14), individual traits (0.12), and knowledge characteristics (0.10). This weighting pattern suggests that classroom-related competencies carry substantially greater importance than other dimensions in the proposed model.

### 5.3. Model Optimization

**Figure 3:**
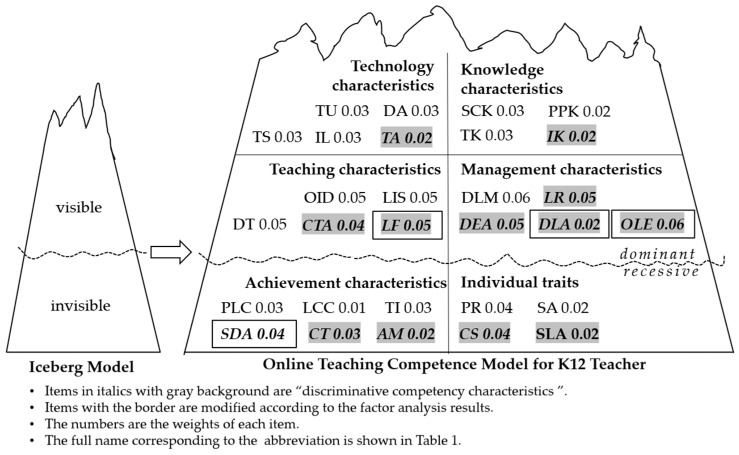
Modified online teaching competency model for K12 teachers.

According to the above factor analysis and weight distribution results, the model was revised and improved (as shown in Figure 3). The above-the-ice surface represented by the wavy line is the explicit competency, and below is the implicit competency; items in italics with gray background are the discriminative competencies, and the others are the benchmark competencies.

## 6. Predictive Relationship Between Online Teaching Competency and Performance (RQ3)

Having established the structural framework of K12 teachers’ online teaching competencies (addressing RQ1 and RQ2), we now turn to RQ3: What is the relationship between the competency characteristics in the model and the prediction of online teaching performance? To test this predictive validity—and simultaneously validate the nomological network of the competency model—we employed structural equation modeling (SEM). The nomological network, the original conceptualization that appeared in Cronbach and Meehl’s (1955) foundational work titled “Construct Validity in Psychological Tests”, requires that antecedent variables or outcome variables must be introduced on the basis of the original model to form the law relationship of the model. This study further verifies the validity of the law relationship between the competency model and the outcome variable (performance) by using the structural equation model.

### 6.1. Research Variables and Hypotheses

In the structural equation model, which explores the relationship between competence and performance, the independent variable is competence, and the dependent variable is performance. Competence, according to the competency model, is expressed by six first-order characteristic factors, including knowledge characteristics, technical characteristics, teaching characteristics, management characteristics, achievement characteristics, and individual traits.

However, there are usually three ways to express performance. First, it is expressed in terms of results or outputs. For example, Bernardin (1984) argued that performance is the record of an individual’s output during a specific job, task, or activity at a specific time. Second, it is expressed in terms of process behavior performance. For example, Campbell (2012) argued that performance is a goal-related behavior controlled by the employees themselves. Obilor (2020) confirmed that high school teachers’ communication skills (one of the competency characteristics) largely affect students’ academic performance (outcomes). Third, it is expressed in terms of behavior and results. For example, Binning and Barrett (1989) argued that the best way to describe performance is to reflect the relationship between behavior and results. This view holds that performance is work-related, multi-dimensional, multi-caused, and dynamically changing. This view is consistent with this study. Stufflebeam (2000) pointed out in the decision-oriented CIPP model that when judging and evaluating the success of a project, not only the outcome factors should be considered, but also the background factors, input factors and process factors should be considered, especially the process factors can provide a lot of effective information for decision makers to revise the project plan. Therefore, in the measurement of online teaching performance, in addition to the outcome performance, this study also emphasizes the achievement behavior of teachers and students in the process of teaching and learning, that is, process performance.

Many scholars have different opinions on the performance or achievement indicators of online teaching and learning. After studying more than ten typical learning analysis systems, Buniyamin et al. (2015) sorted out multiple indicators, including academic performance, course participation, learning style, social performance, etc. When studying learning achievement, Bukralia et al. (2015) used academic ability, economic level, academic goals, technical preparation, course motivation, and participation as predictors. Sun and Fang (2019) analyzed the performance analysis framework of online learning into seven dimensions: engagement level, interaction level, positive level, stage achievement, learning attitude, learning habits, and frustration level. F. X. Guo and Liu (2018) used classroom behavior, homework performance, cognitive level, and test scores as indicators to evaluate learning effects in online learning research based on Blackboard. In addition, there are a large number of studies on online teaching effects, such as SPOC, MOOC, flipped classroom, and mixed classroom, most of which use variables such as academic performance, test pass rate, learning motivation, learning interest, and student satisfaction to represent learning effects.

In the process of online teaching, there are not only students but also teachers. Therefore, the measurement of online teaching performance should not only consider the development of students, but also the development of teachers. Therefore, drawing on Binning and Barrett’s (1989) framework, this study adopts a dual-perspective approach involving both teachers and students. Specifically, it evaluates K12 online teaching performance through two dimensions—process performance and outcome performance—while integrating the perspectives of these two key stakeholders. Process performance is mainly measured from learning habits, knowledge transfer ability, learning interest, self-study learning ability, learning input, etc. Outcome performance is mainly measured from learning achievement, teaching goal achievement, teaching satisfaction, and teacher professional development.

In summary, the following research hypotheses (H) are proposed for exploring the relationship between competence and performance in K12 online teaching.

**H1a.**  *Knowledge competency characteristics have a significant predictive effect on process performance.*

**H1b.**  *Knowledge competency characteristics have a significant predictive effect on outcome performance.*

**H2a.**  *Technical competency characteristics have a significant predictive effect on process performance.*

**H2b.**  *Technical competency characteristics have a significant predictive effect on outcome performance.*

**H3a.**  *Teaching competency characteristics have a significant predictive effect on process performance.*

**H3b.**  *Teaching competency characteristics have a significant predictive effect on outcome performance.*

**H4a.**  *Management competency characteristics have a significant predictive effect on process performance.*

**H4b.**  *Management competency characteristics are significant predictors of outcome performance.*

**H5a.**  *Achievement competency characteristics have a significant predictive effect on process performance.*

**H5b.**  *Achievement competency characteristics have a significant predictive effect on outcome performance.*

**H6a.**  *Personal competency characteristics have a significant predictive effect on process performance.*

**H6b.**  *Personal competency characteristics are significant predictors of outcome performance.*

### 6.2. Research Tool Design and Data Collection

In the context of this study, the measurement items of process performance were adapted from the learning performance and participation in Buniyamin et al. (2015), academic ability in Bukralia et al. (2015), learning habits and learning attitudes in Sun and Fang (2019), cognitive level, knowledge transfer, and learning engagement in F. X. Guo and Liu (2018), and learning interaction, learning interest, and autonomous learning ability in X. Luo (2016). The measurement items of outcome performance were adapted from the teaching satisfaction, teaching professional ability, and self-compiled student achievement and teaching goal achievement in TALIS2018. After completing the design of the measurement indicators of each latent variable, the questionnaire was designed for each measurement dimension in the form of a 5-point Likert scale. The 5-point Likert scale measures attitudes by asking respondents to rate statements on a symmetric agreement scale (1 = strongly disagree, 5 = strongly agree), converting qualitative responses into quantifiable data.

In order to ensure the validity of the final survey data, a pre-survey of the initial questionnaire was carried out before the formal survey. The pre-survey was administered through our established network of collaborating teachers in primary and secondary schools across multiple regions, including Shenzhen, Dongguan, Yongkang, and Wuhan. These participating teachers, with whom we had previous research collaborations, helped distribute the questionnaires, resulting in 162 valid responses. According to the feedback from the pre-survey teachers, the description of the question items was simplified to reduce the cognitive load of the respondents. Finally, a questionnaire composed of 1 screening question item, 11 sample background and characteristic questions (single and multiple-select questions), 50 competence characteristic variable questions, 17 online teaching performance variable questions, a total of 79 question items was formed. To assess teachers’ online teaching performance, this study employed teacher self-reports of observable student outcomes. Given that students were the primary participants in online instruction, teachers evaluated their own effectiveness based on perceived student improvements. For instance, teachers were asked to rate statements such as “Online assignment sharing and feedback improved students’ work quality through peer learning”.

According to the principle of convenient sampling, the questionnaires were distributed in the working group of audio-visual education centers in Hubei Province through the Wenjuanxing, and then forwarded by the teachers of audio-visual education centers in various places to the teachers of local primary and secondary schools. After the survey, a total of 13,865 teacher questionnaires were collected from 41 districts (counties) from the Wenjunxing platform, most of which were filled in through mobile phones by means of WeChat forwarding links. The questionnaires with a response time of less than 200 s were screened out, the samples that participated in online teaching for less than 10 class hours were deleted, and 12,726 valid questionnaires were recovered, with an effective collection rate of 91.78%.

### 6.3. Reliability and Validity Analysis

In order to test the reliability of the questionnaire, this study used the Cronbach’s α coefficient to measure the degree of internal consistency for reliability testing. According to the standard of Nunnally (1978), when the α coefficient is greater than 0.7, the questionnaire has good internal consistency. For the six competence variables and two performance variables in the model, the Cronbach’s α coefficient of the measurement items is greater than 0.7, indicating that the measurement reliability is good.

The core variables of competence were tested by exploratory factor analysis in Section 5.1. The retained factor loads were all greater than 0.5, and the structural validity was good.

Exploratory factor analysis (EFA) was used to test the structural validity of the performance variables measured in the questionnaire. There were 17 performance items in total. The results of KMO and Bartlett spherical tests showed that the KMO was 0.856, greater than 0.7. The approximate chi-square value of the Bartlett spherical test was 11,342.734, the degree of freedom (df) was 336, and the significance (Sig.) was 0.000. This shows that the data concentration of each performance item is good, which is suitable for factor analysis.

Next, the principal component method was used to extract the factors. The eigenvalues were greater than 1, and the maximum variance method was used to rotate, sort by size, and the coefficients of less than 0.5 were cancelled. After orthogonal rotation, a total of two common factors were extracted, and the cumulative variance rate is 74.405%, indicating that the extracted two common factors and 17 items can well explain the performance variable information (as shown in Table 7). The rotated factor loads are all greater than 0.6, indicating that the structural validity of the performance measurement items is good.

### 6.4. Path Analysis and Hypothesis Testing

The online teaching competency of the surveyed teachers was rated at an upper-middle level across six dimensions (measured on a 5-point Likert scale ranging from 1 [strongly disagree] to 5 [strongly agree]). Specifically, the mean scores were as follows: cognitive characteristics (M = 3.509), technical characteristics (M = 3.496), teaching characteristics (M = 3.570), management characteristics (M = 3.562), achievement characteristics (M = 3.544), and individual traits (M = 3.574). Notably, all participants in this study were teachers with prior experience in online instruction. Their sustained engagement in professional training and practical implementation of online teaching over recent years contributed to their relatively high overall competency. Both process performance (M = 3.623) and outcome performance (M = 3.235) were similarly positioned within the upper-middle range of the scale (1–5 points). Among them, the process performance is significantly higher than the result performance, which also shows that the implicit performance in the online teaching process in terms of learners’ learning habits, interests, and investment cannot be ignored.

The structural model analysis yielded nuanced insights into competency-performance relationships. Among the 12 hypothesized paths, 10 demonstrated statistical significance (*p* < 0.05), confirming the overall model validity (Table 8). However, two non-significant relationships warrant attention: (1) knowledge characteristics → process performance (*p* = 0.178 > 0.05) and (2) individual traits → outcome performance (*p* = 0.527 > 0.05). That is to say, the prediction effect of knowledge competency characteristics on online teaching process performance is not significant; the competence of individual traits dimension has no significant effect on online teaching outcome performance prediction. These exceptions suggest that knowledge application in online teaching may depend more on pedagogical integration (technical/instructional competencies) than pure content knowledge, and personality factors might mediate—rather than directly determine—outcomes, aligning with Bandura and Wessels’s (1997) social cognitive theory of reciprocal determinism.

The joint variability interpretation rate of each dimension of competency and process performance was 54.8%, and the joint variability interpretation rate of outcome performance was 45.2%. It is remarkably high for behavioral research. The management competency characteristics had the strongest predictive effect on process performance (β = 0.363) and outcome performance (β = 0.289). It emphasized the centrality of organizational skills in virtual classrooms and the need to prioritize structured facilitation over purely technical training in teacher development programs.

### 6.5. Fitting Test of Structural Models

The results of the fitting degree parameters of the structural model of competence and performance shown in Table 9 show that the chi-square–degree of freedom ratio χ^2^/df is 1.927, which is less than 3; the GFI is 0.913, the AGFI is 0.911, the CFI is 0.917, the IF I is 0.920, and the TLI is 0.912, which are all greater than the fitting standard 0.9; the SRMR is 0.043, which satisfies the fitting standard less than 0.05; the RMSEA is 0.048, which satisfies the fitting standard less than 0.08. It can be considered that the structural model of the relationship between competence and performance has a good fit, indicating that the path relationship proposed in this study is in good agreement with the actual measurement data.

## 7. Conclusions and Application Recommendations

### 7.1. Conclusions

This study establishes a comprehensive online teaching competency model for K12 teachers, consisting of six core dimensions: knowledge characteristics, technical characteristics, instructional characteristics, management characteristics, achievement characteristics, and individual traits. These dimensions collectively encompass 29 competency elements, with 12 serving as discriminative features and 17 as generic characteristics. Notably, the first four dimensions represent explicit competencies, while achievement characteristics and individual traits reflect implicit qualities.

The proposed model demonstrates a robust multidimensional hierarchical structure with satisfactory goodness-of-fit indices. While exploratory factor analysis confirmed the initial conceptualization of knowledge characteristics and technical characteristics, other dimensions required model adjustments based on empirical findings.

The competency-performance structural analysis revealed strong model fit, with 10 of the 12 hypothesized paths showing statistical significance. Two exceptions were the nonsignificant paths from knowledge characteristics to process performance (*p* = 0.178) and from individual traits to outcome performance (*p* = 0.527). These results generally support our theoretical framework linking teacher competencies to online teaching performance.

In addition, this study has several limitations that should be acknowledged. First, as participants’ online teaching experiences were conducted across different platforms rather than a unified system, platform-specific characteristics may have influenced the results. Second, while we examined the relationship between online teaching competence and performance, the analysis did not account for potential mediating or moderating effects that could provide deeper insights into this relationship.

Looking ahead, the ongoing development of China’s National Smart Education Platform presents valuable opportunities for future research. This standardized platform could facilitate more controlled investigations of online teaching competencies while eliminating platform-related variances. Additionally, future studies could explore mediating mechanisms (e.g., teachers’ technological pedagogical knowledge) and moderating factors (e.g., school support) that may influence the competence-performance relationship.

### 7.2. Application Recommendations for the Online Teaching Competency Model

#### 7.2.1. Designing Competency-Based Online Teaching Norms for K12 Educators

Current online teaching norms for K12 educators predominantly emphasize technical operations and explicit skills (e.g., platform navigation, digital resource management), as noted by B. X. Guo (2015). However, findings from this study highlight the critical role of implicit competencies—such as adaptive awareness of student needs (achievement characteristics) and collaborative resource-sharing behaviors (individual traits)—in shaping online teaching performance.

To address this gap, we propose developing comprehensive teaching standards grounded in the six-dimensional competency model (knowledge, technical, instructional, management, achievement, and individual traits). These norms should systematically integrate observable behavioral benchmarks across all phases of online instruction, from lesson preparation to post-teaching evaluation.

For explicit competencies, norms could standardize technical protocols (e.g., “Apply interactive whiteboard tools to facilitate real-time student engagement”) and pedagogical practices (e.g., “Design asynchronous discussion boards aligned with lesson objectives”). Meanwhile, implicit competencies require innovative assessment strategies, such as evaluating teachers’ ability to personalize instruction based on learning analytics or their participation in peer-driven resource networks.

To ensure developmental progression, norms should adopt a tiered structure: foundational standards might focus on platform mastery (technical characteristics), intermediate tiers on data-informed instructional adjustments (instructional + achievement characteristics), and advanced tiers on fostering collaborative learning ecosystems (management + individual traits).

Iterative refinement of these standards should leverage the competency-performance pathways identified in this study, particularly prioritizing dimensions with higher explanatory power (e.g., 54.8% variance in process performance).

#### 7.2.2. Competency Model-Driven Online Teacher Training for K12 Educators

Teacher training programs, while critical for professional development, often operate under the flawed assumption that knowledge and skill acquisition directly translate to teaching competence—an approach that overlooks the complex cognitive processes and adaptive problem solving that are inherent to effective pedagogy. To address this limitation, we suggest that the training framework can be aligned with the six-dimensional competence model of online teaching. This paradigm shift prioritizes performance-oriented training, emphasizing how teachers integrate competencies to resolve real-world instructional challenges rather than merely mastering discrete skills.

The framework differentiates training strategies based on competency typology and plasticity. The explicit competency characteristics are easier to acquire, while the implicit competency characteristics require a long time period; the benchmark competency characteristics are easy to improve quickly through training, while the discriminative competency characteristics are difficult to change through short-term training. Therefore, the appropriate training type and priority should be determined according to the attributes and weights of different competency characteristics, for instance, by allocating 40% of training program hours to high-impact management and achievement characteristics. Teachers already have a knowledge base in pre-service education, so the knowledge characteristics can be trained by borrowing systematic training resources. While the collaborative teaching ability, data-based learning situation analysis, online learning evaluation, etc. in the teaching characteristics belong to the discriminative competence characteristics, and the weight is high, so the selection of backbone teacher training can be carried out; for the information technology skills that can be quickly acquired, it can be completed through school-based short-term internal training.

#### 7.2.3. Competency Model-Informed Evaluation Strategies for K12 Online Teaching

The competency model is the behavioral operationalization of online teaching, encompassing explicit dimensions (knowledge, technical, instructional, management) and implicit qualities (achievement, individual traits). It provides a robust framework for designing multidimensional teacher evaluation systems. Traditional assessment practices often conflate observable technical proficiency with holistic teaching competence, thereby undervaluing critical but latent capacities such as adaptive student engagement (achievement characteristics) or collaborative innovation (individual traits).

For explicit competencies, standardized quantitative rubrics can objectively assess skills such as lesson structure coherence (instructional characteristics) or platform functionality mastery (technical characteristics). Conversely, implicit competencies demand many different methods. For example, structured classroom observations could capture teachers’ ability to personalize instruction based on real-time analytics (achievement characteristics). Crucially, the 54.8% variance explained in process performance suggests embedding longitudinal assessments to track competency development trajectories, such as semester-long case studies analyzing teachers’ responsiveness to student engagement data.

In addition, weight allocation should mirror the model’s structural hierarchy. For example, assigning 30% of evaluation scores to management characteristics, given their outsized impact on both process and outcome performance.

## Figures and Tables

**Table 1:** Interview outline according to the STAR tool.

Situation/Task	Action	Result
What was the situation of the “success/regret” event of online teaching you want to tell about? What tasks did you face, and what goals did you want to achieve?	Who participated in your task? What measures did you take to complete the task? How did you judge, or what are the reasons that made you choose this measure? What difficulties did you encounter in completing the task and how did you solve them?	What was the outcome or effect of the task completed in this event? What do you think were the main factors that influenced this outcome or effect?

**Table 2:** Open coding nodes from the interview texts.

Competency Characteristics of Online Teaching ^1^			
subject content knowledge	SCK	learner retention	LR
pedagogical knowledge	PK	distance emotional awareness	DEA
psychological knowledge	PSK	distance learning monitoring	DLM
technical knowledge	TK	individual learning feedback	ILF
integrated knowledge	IK	group learning feedback	GLF
technology selection	TS	personalized learning concept	PLC
technology adaptation	TA	learning-centered concept	LCC
technology use	TU	teaching innovation	TI
data awareness	DA	community thinking	CT
information literacy	IL	achievement motivation	AM
distance teaching ability	DT	teaching self-efficacy	TSE
collaborative teaching ability	CTA	self-development awareness	SDA
online interaction design	OID	self-learning ability	SLA
data-based learning analysis	DLA	communication skills	CS
online learning evaluation	OLE	professional responsibility	PR
learning intervention strategies	LIS	social adaptability	SA

^1^ The capital letters after the items are abbreviations. For example, achievement motivation is abbreviated as AM.

**Table 3:** Six core genera.

Core Genera	Competency Characteristics
Knowledge characteristics	SCK, pedagogical and psychological knowledge (PPK), TK, FK
Technology characteristics	TS, TA, TU, DA, IL
Teaching characteristics	DT, CTA, OID, DLA, OLE, LIS
Management characteristics	LR, DEA, DLM, learning feedback (LF)
Achievement characteristics	PLC, LCC, TI, CT, AM
Individual traits	TSE, SDA, CS, PR, SA

**Table 4:** Results of paired *t*-test analysis ^1^.

Characteristics	High-Performance Group (*n* = 12)		Ordinary-Performance Group (*n* = 10)		t	Sig.
	Mean	S.D.	Mean	S.D.		
SCK	4.410	0.831	4.176	0.622	0.551	0.587
PK	3.306	0.650	3.248	0.682	1.930	0.067
PSK	3.013	0.686	3.215	0.603	1.763	0.117
TK	3.711	0.641	3.548	0.612	1.929	0.067
**IK**	**3.839**	**0.735**	**3.253**	**0.618**	**1.975**	0.047 *
TS	3.829	0.848	3.368	0.601	1.251	0.224
**TA**	**3.745**	**0.776**	**3.376**	**0.690**	**3.304**	0.003 ***
TU	3.918	0.848	3.280	0.650	2.227	0.036
DA	4.127	0.722	4.066	0.600	0.474	0.640
IL	4.061	0.702	3.348	0.598	0.343	0.735
DT	4.220	0.846	3.533	0.665	0.977	0.339
**CTA**	**3.818**	**0.820**	**3.374**	**0.622**	**3.285**	0.003 **
**OID**	3.484	0.864	3.270	0.645	1.269	0.218
**DLA**	**3.252**	**0.719**	**3.113**	**0.635**	**2.827**	0.027 *
**OLE**	**4.227**	**0.760**	**3.097**	**0.629**	**2.707**	0.031 **
LIS	3.205	0.807	3.001	0.630	0.008	0.994
**LR**	**3.846**	**0.723**	**3.222**	**0.649**	**3.700**	0.003 **
**DEA**	**3.821**	**0.823**	**3.310**	**0.660**	**2.529**	0.019 **
DLM	3.674	0.865	3.468	0.690	1.246	0.226
**ILF**	**3.418**	**0.864**	**3.371**	**0.634**	**3.247**	0.004 **
PLC	3.765	0.669	3.269	0.615	2.216	0.045
LCC	3.627	0.626	3.241	0.629	1.861	0.076
TI	3.739	0.797	3.209	0.618	1.586	0.127
**CT**	**3.816**	**0.873**	**3.461**	**0.663**	**3.673**	0.000 ***
**AM**	**4.025**	**0.832**	**3.650**	**0.640**	**4.167**	0.000 ***
**TSE**	**4.158**	**0.864**	**3.461**	**0.642**	**4.453**	0.000 ***
SDA	3.736	0.780	3.301	0.582	0.008	0.994
**CS**	**3.528**	**0.747**	**3.168**	**0.573**	**4.246**	0.000 **
PR	3.016	0.831	2.808	0.622	1.579	0.129
SA	4.112	0.650	4.004	0.682	1.529	0.117

^1^ The bolded rows in the table emphasize items demonstrating statistically significant differences between the two groups. * *p* < 0.05, ** *p* < 0.01,*** *p* < 0.001.

**Table 5:** Sample distribution.

Sample Distribution		Frequency	Percentage (%)
School Location	Provincial Capital	1217	27.8
	Prefecture-level City	1192	27.2
	County/District	1387	31.7
	Township/Rural Area	582	13.3
Gender	Male	1603	36.3
	Female	2775	63.7
Age	Below 30	1109	25.3
	31–40	993	22.7
	41–50	1715	39.2
	Above 50	561	12.8
Teaching years	Below 5 Years	1132	17.3
	6–10 Years	525	9.8
	11–15 Years	657	4.8
	Above 15 Years	2064	68.1
Education	Associate Degree or Below	1737	24.6
	Bachelor’s Degree	2492	73.0
	Master’s Degree or Above	149	2.5
Teaching Level	Primary School	2711	62.8
	Junior High School	1179	28.2
	Senior High School	488	9.0
Professional Title	Senior Teacher	746	17.0
	First-Level Teacher	1376	31.4
	Second-Level Teacher	1032	23.6
	Third-Level Teacher/No title	1224	28
Role in online learning	Online Q&A/Support Teachers	1357	31.0
	Live/Recorded Lecture Teachers	1954	44.6
	Both roles	1067	24.4
Total		4378	100.0

**Table 6:** Competency factor weight analysis results.

Core Genera	Characteristics	Coefficients in Linear Combinations (Factor Weights)						Score in the Model	Secondary Weight	First-Level Weight
		1	2	3	4	5	6			
Teaching	OID	0.21	0.02	0.02	0.14	0.06	−0.12	0.11	0.05	0.24
	LIS	0.19	0.06	0.01	0.03	0.05	0.09	0.11	0.05	
	DT	0.19	0.06	0.01	0.03	0.05	0.04	0.10	0.05	
	LF	0.16	0.06	0.01	0.10	−0.02	0.07	0.09	0.05	
	CTA	0.15	0.06	0.01	0.11	0.02	−0.04	0.09	0.04	
Knowledge	TK	0.05	0.06	0.05	0.08	0.01	0.06	0.05	0.03	0.10
	IK	0.04	0.05	0.05	0.10	−0.01	0.07	0.05	0.02	
	SCK	0.04	0.06	0.09	0.08	0.01	−0.01	0.05	0.03	
	PPK	0.04	0.05	0.02	0.10	−0.02	0.07	0.04	0.02	
Technology	IL	0.01	0.05	0.35	−0.09	0.06	0.11	0.07	0.03	0.14
	TA	0.01	0.05	0.29	−0.07	0.07	−0.14	0.05	0.02	
	DA	0.01	0.05	0.28	−0.07	0.08	0.16	0.06	0.03	
	TU	0.00	0.06	0.28	0.07	−0.08	0.18	0.06	0.03	
	TS	0.00	0.06	0.24	0.06	0.03	0.09	0.06	0.03	
Achievement	AM	0.03	0.06	−0.01	0.07	−0.09	0.20	0.04	0.02	0.16
	LCC	0.03	−0.06	0.00	0.07	0.02	−0.05	0.01	0.01	
	TI	0.08	0.06	0.01	0.16	0.02	−0.01	0.07	0.03	
	PLC	0.09	0.07	−0.01	0.18	−0.02	0.06	0.07	0.03	
	SDA	0.08	0.06	0.00	0.16	−0.01	0.03	0.07	0.03	
	CT	0.09	0.06	−0.03	0.18	−0.05	0.12	0.07	0.03	
Management	LR	0.08	0.19	0.10	0.01	0.21	−0.07	0.10	0.05	0.24
	DEA	0.05	0.27	0.09	0.00	0.13	0.07	0.11	0.05	
	OLE	0.06	0.27	0.16	0.01	0.16	0.08	0.12	0.06	
	DLM	0.05	0.26	0.16	0.02	0.13	0.08	0.12	0.06	
	DLA	0.00	0.24	−0.15	0.01	0.01	0.01	0.04	0.02	
Individual	TSE	0.00	0.24	−0.10	0.01	0.01	0.01	0.05	0.02	0.12
	PR	0.05	0.24	0.11	0.01	−0.28	0.14	0.08	0.04	
	CS	0.01	0.23	0.19	−0.01	0.01	0.03	0.09	0.04	
	SA	0.01	0.23	−0.21	0.01	0.02	0.03	0.03	0.02	

**Table 7:** Component matrix after performance measurement item rotation ^1,2^.

Title 1	Title 2	Title 3
PP Item 5: Learning habits	0.876	
PP Item 8: Communication and collaboration	0.871	
PP Item 4: Autonomous learning ability	0.860	
PP Item 7: Liking to learn	0.859	
PP Item 6: Enthusiasm	0.849	
PP Item 3: Interest in learning	0.843	
PP Item 1: Assignment	0.755	
PP Item 2: Knowledge transfer	0.747	
OP Item 8: Teaching capacity enhancement		0.897
OP Item 6: Technology improvement		0.863
OP Item 7: Resource accumulation		0.833
OP Item 1: Learning achievement		0.720
OP Item 2: Knowledge mastery		0.719
OP Item 3: Learning needs satisfied		0.697
OP Item 4: Learning objectives achieved		0.690
OP Item 9: Continuous use intention		0.688
RP Item 5: Satisfaction		0.610
Eigenvalue	10.612	2.037
Explanation rate of variance	41.585	32.820
Cumulative variance interpretation rate	41.585	74.405

^1^ Extraction method: principal component analysis. Rotation method: Caesar normalized maximum variance method. The rotation has converged after three iterations. ^2^ PP: Process performance; OP: Outcome performance.

**Table 8:** Path analysis results ^1^.

Hypothesis	Path Relationships			Estimate	S.E.	C.R.	*p*	Standardized Path Coefficient (β)	Result
H1a	PP	<---	Knowledge characteristics	0.089	0.066	1.348	0.178	0.079	Unsupported
H1b	OP	<---	Knowledge characteristics	0.282	0.018	15.667	***	0.265	Supported
H2a	PP	<---	Technology characteristics	0.201	0.011	18.273	***	0.192	Supported
H2b	OP	<---	Technology characteristics	0.191	0.019	10.053	***	0.178	Supported
H3a	PP	<---	Teaching characteristics	0.086	0.011	7.818	***	0.072	Supported
H3b	OP	<---	Teaching characteristics	0.272	0.013	20.923	***	0.268	Supported
H4a	PP	<---	Management characteristics	0.264	0.018	14.667	***	0.289	Supported
H4b	OP	<---	Management characteristics	0.372	0.027	13.778	***	0.363	Supported
H5a	PP	<---	Achievement characteristics	0.161	0.012	13.417	***	0.146	Supported
H5b	OP	<---	Achievement characteristics	0.182	0.017	10.706	***	0.165	Supported
H6a	PP	<---	Individual traits	0.052	0.032	1.625	0.527	0.047	Unsupported
H6b	OP	<---	Individual traits	0.124	0.011	11.273	***	0.109	Supported

^1^ *** *p* < 0.001. PP: Process performance; OP: Outcome performance.

**Table 9:** Relationship between competency and performance, structural model fitting index.

Statistical Test	Absolute Fit Index					Relative Fit Index		
	χ^2^/df	GFI	AGFI	SRMR	RMSEA	CFI	IFI	TLI
Adaptation standard	<3	>0.9	>0.9	<0.05	<0.08	>0.9	>0.9	>0.9
Parameters of this model	1.927	0.913	0.911	0.043	0.048	0.917	0.920	0.912
Other parameters: χ^2^ = 1882.679; *p* = 0.000; df = 977								

## Data Availability

Data are available on request due to restrictions. The data presented in this study are available on request from the corresponding author for two reasons. First, the data are not publicly available due to privacy restrictions such as school, age, teaching subject, etc. Second, the data are part of an ongoing longitudinal study. Full public release would compromise ongoing research objectives.
